# Optical characterization of porous silicon monolayers decorated with hydrogel microspheres

**DOI:** 10.1186/1556-276X-9-425

**Published:** 2014-08-22

**Authors:** Ruth F Balderas-Valadez, Markus Weiler, Vivechana Agarwal, Claudia Pacholski

**Affiliations:** 1 CIICAp, UAEM, Av., Universidad 1001 Col. Chamilpa, Cuernavaca, Morelos 62210, Mexico; 2 Department of New Materials and Biosystems, Max Planck Institute for Intelligent Systems, Heisenbergstr. 3, Stuttgart 70569, Germany; 3 Department of Biophysical Chemistry, University of Heidelberg, Im Neuenheimer Feld 253, Heidelberg 69120, Germany

**Keywords:** Porous silicon, Hydrogel, Self-assembly, Sensor

## Abstract

The optical response of porous silicon (pSi) films, covered with a quasi-hexagonal array of hydrogel microspheres, to immersion in ethanol/water mixtures was investigated. For this study, pSi monolayers were fabricated by electrochemical etching, stabilized by thermal oxidation, and decorated with hydrogel microspheres using spin coating. Reflectance spectra of pSi samples with and without deposited hydrogel microspheres were taken at normal incidence. The employed hydrogel microspheres, composed of poly-N-isopropylacrylamide (polyNIPAM), are stimuli-responsive and change their size as well as their refractive index upon exposure to alcohol/water mixtures. Hence, distinct differences in the interference pattern of bare pSi films and pSi layers covered with polyNIPAM spheres could be observed upon their immersion in the respective solutions using reflective interferometric Fourier transform spectroscopy (RIFTS). Here, the amount of reflected light (fast Fourier transform (FFT) amplitude), which corresponds to the refractive index contrast and light scattering at the pSi film interfaces, showed distinct differences for the two fabricated samples. Whereas the FFT amplitude of the bare porous silicon film followed the changes in the refractive index of the surrounding medium, the FFT amplitude of the pSi/polyNIPAM structure depended on the swelling/shrinking of the attached hydrogel spheres and exhibited a minimum in ethanol-water mixtures with 20 wt% ethanol. At this value, the polyNIPAM microgel is collapsed to its minimum size. In contrast, the effective optical thickness, which reflects the effective refractive index of the porous layer, was not influenced by the attached hydrogel spheres.

**PACS:**

81.05.Rm; 81.16.Dn; 83.80Kn; 42.79.Pw

## Background

Porous silicon (pSi) is a well-established material for the tailor-made fabrication of optical biosensors and can be easily prepared by electrochemical etching. The simplicity of its fabrication process in combination with its intrinsic large surface area and convenient surface chemistry has considerably pushed this research field. The optical transduction in pSi sensors is based on changes in the interference pattern which results from the reflection of light at the interfaces of the porous silicon film. To improve the sensitivity of pSi sensors, more sophisticated optical structures such as rugate filters, Bragg reflectors, and microcavities have been realized by modulating the porosities of the pSi using appropriate etching parameters. These structures possess peaks with narrow bandwidths in their reflectance spectra, and consequently, they are more sensitive in comparison to pSi monolayers showing Fabry-Pérot interference patterns [1,2]. Another route to highly sensitive optical pSi sensors is the introduction of a diffraction grating into the porous material [3-6].

Besides the tremendous progress in the optimization of the optical properties of pSi sensors, other challenges such as the stability of the pSi films in basic aqueous solutions and efficient surface functionalization have been heavily investigated [7]. A very promising and intriguing approach to further improve the performance of porous silicon sensors is the integration of polymers [8]. For this purpose, different strategies have been tested, including coating of the porous silicon layer with a polymer film [9], infiltration of polymer into the porous matrix [10,11], and polymer microdroplet patterning of porous silicon structures [12]. The fabricated polymer/porous silicon hybrids showed a better stability in aqueous biological media and considerably improved sensitivity in optical biosensing experiments in comparison to unmodified porous silicon. Especially the combination of porous silicon with a special class of polymers, namely hydrogels, has led to this progress [13-15]. Hydrogels are hydrophilic polymeric networks which are characterized by their stimuli-responsive properties. Depending on their chemical composition and internal structure, hydrogels react sensitively to external triggers such as temperature, pH, and ionic strength, which cause abrupt volume changes in the hydrogel. This volume change is accompanied by a change in the refractive index of the hydrogel [16]. Hence, the foundation for successfully utilizing hydrogels for the fabrication of highly sensitive optical sensors is a reasonable understanding of the influence of the volume change on the thickness as well as the refractive index of the hydrogel and their impact on the optical response of the sensor.

We envision an optical sensor composed of a highly ordered array of hydrogel microspheres on top of a porous silicon film. This sensor will offer two different ways of optical transduction: scattering/diffraction of light resulting from the deposited array of hydrogel microspheres and interference of light rays reflected at the interfaces of the porous silicon film. In this work, we will report on the fabrication of porous silicon monolayers covered with a non-close packed array of hydrogel microspheres and their optical properties in comparison to bare porous silicon films.

## Methods

Silicon wafers (p-type, boron doped, <100 > orientation, resistivity ≤ 0.001 Ω cm) were obtained from Siltronix Corp. (Archamps, France). Hydrofluoric acid (HF), ethanol, and H_2_O_2_ were supplied by (Merck KGaA, Darmstadt, Germany). *N*-isopropylacrylamide (NIPAM) and 3-aminopropyltriethoxysilane (APTES) were purchased from Sigma-Aldrich Chemie GmbH (Munich, Germany). *N*,*N*′-methylenebisacrylamide (BIS), H_2_SO_4_, and HCl were received from Carl Roth (Karlsruhe, Germany). Potassium peroxodisulfate (KPS) was supplied by Fluka (St. Louis, MO, USA). Water was deionized to a resistance of at least 18.2 MΩ (Ultra pure water system (TKA, Niederelbert, Germany)) and then filtered through a 0.2-μm filter.

Scanning electron microscopy (SEM) images were obtained with a Zeiss Ultra 55 ‘Gemini’ scanning electron microscope (Carl Zeiss, Inc., Oberkochen, Germany) using an accelerating voltage of 3 keV and an in-lens detector. To suppress charging of the sample during imaging, the samples were coated with carbon prior to SEM analysis using a Bal-Tec MED 020 sputter coater (Bal-Tec AG, Balzers, Liechtenstein).

Reflectance spectra were recorded at normal incidence using an Ocean Optics charge-coupled device (CCD) spectrometer (Ocean Optics GmbH, Ostfildern, Germany) fitted with a microscope objective lens connected to a bifurcated fiber optic cable. A tungsten halogen light source was focused on the sample surface with a spot size of approximately 2 mm^2^. Reflectance data were collected with a CCD detector in the wavelength range of 500 to 1,000 nm. Experimental reflectance spectra were analyzed by applying a fast Fourier transform (FFT) using the software IGOR Pro (http://www.wavemetrics.com). Details of the analysis can be found in [17]. In order to allow for a direct comparison of the effective optical thickness (EOT) values and FFT amplitude values from different pSi samples, all FFT spectra were normalized by setting the highest value equal to 1 and the lowest value equal to 0.

Dynamic light scattering (DLS) measurements were carried out with a Malvern Instruments Zetasizer Nano ZS (Malvern Instruments, Malvern, UK). Refractive indices, dielectric constants, and viscosities of the ethanol/water mixtures were taken from literature [18,19].

Atomic force microscopy (AFM) images were obtained with a JPK Nanowizard II (JPK Instruments AG, Berlin, Germany) in intermittent contact mode (cantilever: Veeco NP-S10, Plainview, NY, USA). Studies on the swelling behavior of the polyNIPAM spheres, attached to the porous silicon surface, were performed in liquid.

### PSi fabrication

Si substrates were cleaned prior to etching by removal of a sacrificial layer of pSi with a strong base. For this purpose, Si substrates were anodized in a solution composed of 3:1 aqueous HF (48 %)/ethanol at 100 mA for 20 s. The resulting porous layer was removed by immersion in a 1 M KOH solution for several minutes. Then, the Si samples were rinsed with ethanol and immersed a second time in a 3:1 aqueous HF (48 %)/ethanol electrolyte. PSi monolayers were formed by electrochemically etching at 100 mA for 5 min. The resulting pSi was rinsed with ethanol and blown dry in a stream of nitrogen. To stabilize the pSi, the samples were oxidized at 300°C for 1 h in an oven.

### PolyNIPAM microsphere synthesis

PolyNIPAM microspheres were prepared by an aqueous free-radical precipitation polymerization according to Pelton and Chibante [20]. Briefly, 0.19 mol/L NIPAM and 0.05 mol/L BIS were dissolved in 124-mL deionized water (approximately 18.2 MΩ cm). The solution was heated to approximately 70°C under inert atmosphere and stirring. Potassium peroxodisulfate (KPS) solution (0.002 mol/L) was added to start the polymerization, which continued for 6 h at approximately 70°C. The resulting polyNIPAM microspheres were purified by subsequent centrifugation, decantation, and redispersion in deionized water. The dispersion was finally filtered (Acrodisc 25-mm syringe filters with Versapor membranes (Pall GmbH, Dreieich, Germany), pore diameter 1.2 μm) and diluted 1:25 (v/v) with deionized water.

### Deposition of polyNIPAM spheres onto pSi

Non-close packed arrays of hydrogel microspheres were deposited on pSi surfaces according to Quint and Pacholski [21]. Briefly, 60 μL of the diluted polyNIPAM dispersion was placed on the oxidized pSi monolayer. To support the formation of an ordered array, 5 μL of ethanol was added and mechanical force was applied by directing a stream of nitrogen to the substrate surface. Finally, the sample was spin-coated at 500 rpm for 6 min (spin coater: Laurell Technologies Corporation, North Wales, PA, USA; model: WS-400B-6NPP/LITE).

The polyNIPAM microspheres were fixed to the surface by silanization. For this purpose, the samples were treated with APTES vapor for 30 min and afterwards baked at 80°C for 1 h.

## Results and discussion

**Figure 1:**
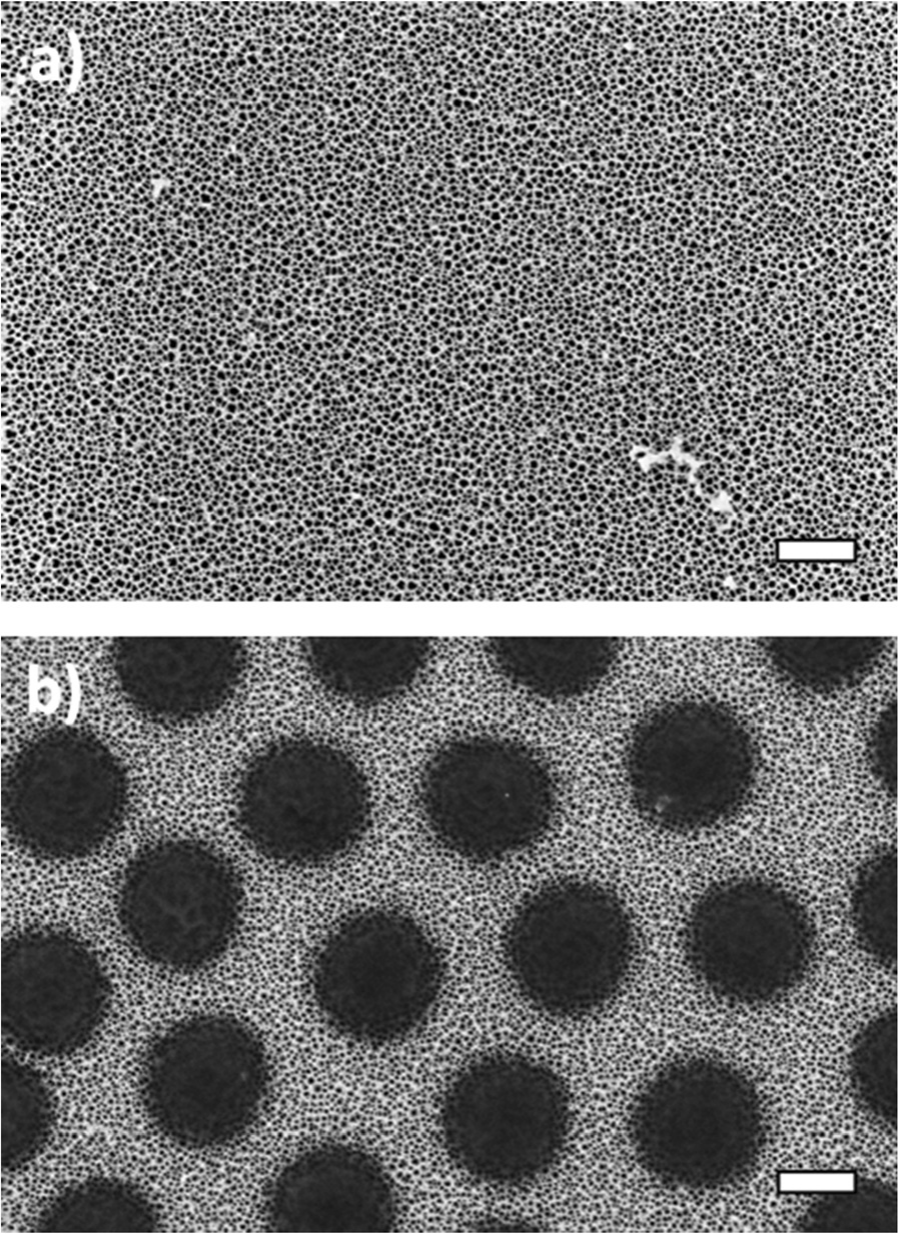
**SEM images of the investigated structures. (a)** pSi monolayer and **(b)** pSi monolayer with a non-close packed array of polyNIPAM microspheres on top. Scale bars, 500 nm.

In Figure 1a,b, SEM images of a bare pSi film as well as a pSi film covered with polyNIPAM microspheres, taken at high magnification, are displayed. SEM images taken at low magnification can be found in Additional file 1: Figure S1. High-magnification SEM images reveal that both porous layers have open pores. The polyNIPAM spheres appear as black circles and form a quasi-hexagonally non-close packed array on top of the pSi layer, whose geometrical arrangement was analyzed with the software package ImageJ. Of the porous surface, 42 ± 3% was covered with hydrogel spheres with a diameter of 837 ± 17 nm and a center to center distance of 1,032 ± 175 nm. The chosen fabrication parameters for the pSi film resulted in a pSi layer thickness of 1,503 ± 334 nm, determined from cross-sectional SEM images, and a porosity of 65 ± 9%, obtained by using the spectroscopic liquid infiltration method (SLIM) [22].

In order to study the influence of the polyNIPAM microspheres on the optical properties of the pSi layer, interferometric reflectance spectra of porous silicon films with and without polyNIPAM spheres were taken at normal incidence. The fringe patterns, observed in the reflectance spectra, result from the interference of reflected light rays at the boundaries of the pSi film, and the position of the fringe maxima can be calculated using the Fabry-Pérot equation:

(1) mλ=2nL

where *m* is an integer, *λ* is the wavelength of the incident light, *n* is the effective refractive index of the pSi film, and *L* is its thickness. By applying a fast Fourier transform to the reflectance spectra, the effective optical thicknesses (EOTs, 2 *nL*) of the porous structures can be directly extracted from the position of the resulting single peak in the frequency spectrum. Changes in the position and amplitude of the FFT peak provide information on the effective refractive index of the pSi layer and the appearance of the involved interfaces, respectively. Hence, a variation in the EOT documents the infiltration of the surrounding medium into the porous layer, and an increase or decrease of the FFT peak indicates variations in the appearance of the porous silicon interfaces, including refractive index contrast and light scattering. This method is referred to as reflective interferometric Fourier transform spectroscopy (RIFTS) [17].

The focus of our investigations was on changes in the reflectance spectra, caused by an external trigger which induces swelling or shrinking of the hydrogel. For this purpose, mixtures of ethanol/water were employed, as polyNIPAM reacts sensitively to their composition. This behavior was explained by cononsolvency which is related to the formation of locally ordered water structures, so-called clathrate structures, resulting from the encapsulation of alcohol molecules by water molecules in alcohol/water mixtures. Hence, the proportion of clathrate structures in the solvent mixture determines the swelling of the hydrogel spheres as they provoke a ‘dehydration’ of the polymer network [23].

**Figure 2:**
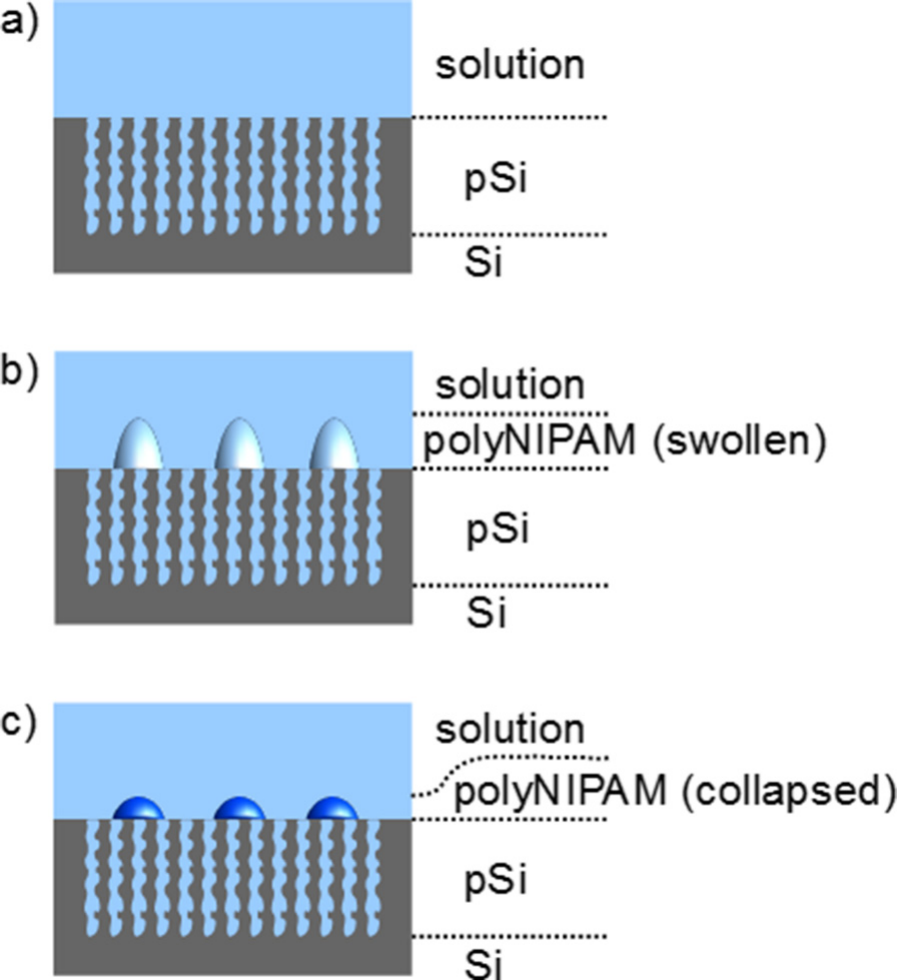
**Illustration of the three investigated structures. (a)** pSi monolayer immersed in water, **(b)** pSi film decorated with swollen polyNIPAM spheres in water, and **(c)** pSi film decorated with collapsed polyNIPAM spheres in water/ethanol mixture (20 wt% ethanol).

Figure 2 illustrates the three most prominent states of the investigated pSi-based structures: a pSi monolayer immersed in water (Figure 2a) and a pSi monolayer decorated with polyNIPAM microspheres which are either in a swollen (Figure 2b) or collapsed (Figure 2c) state, depending on the composition of the surrounding medium. The reference sample, composed of a pSi monolayer, showed a typical Fabry-Pérot interference pattern in its reflectance spectrum. The corresponding FFT was characterized by a single peak whose position is dictated by the effective refractive index of the porous layer. Its amplitude reflects the refractive index contrast at the pSi interfaces in combination with light-scattering events at the pSi/solution interface. Deposition of polyNIPAM spheres onto the pSi film (Figure 2b,c) should result in a more complicated interference pattern, originating from reflection of light at three interfaces: solution/polyNIPAM spheres, polyNIPAM spheres/pSi, and pSi/Si. This would theoretically lead to the appearance of three peaks in the FFT spectra which are related to layer 1 (polyNIPAM spheres), layer 2 (pSi film), and layer 3 (polyNIPAM spheres + pSi film). The reflectance spectrum can be described by a double layer interference model (Equation 2) [17,24]. This model neglects multiple reflections and light scattering:

(2) $$\begin{array}{ll} R=\left[\rho_{a}^{2}+\rho_{b}^{2}+\rho_{c}^{2}\right] & +2\rho_{a}\rho_{b}\cos\left(2d_{\mathrm{pSi}}\right)\\ & +2\rho_{b}\rho_{c}\cos\left(2d_{\mathrm{polyNIPAM}}\right)\\ & +2\rho_{a}\rho_{c}\cos\left(2\left(d_{\mathrm{pSi}}+d_{\mathrm{polyNIPAM}}\right)\right) \end{array}$$

The employed phase relationships *d*_pSi_ and *d*_polyNIPAM_ can be described by Equations 3 and 4:

(3) $$d_{\mathrm{pSi}}=2\pi n_{\mathrm{pSi}}L_{\mathrm{pSi}}/\lambda$$

and

(4) $$d_{\mathrm{polyNIPAM}}=2\pi n_{\mathrm{polyNIPAM}}L_{\mathrm{polyNIPAM}}/\lambda$$

where *n*_pSi_ and *n*_polyNIPAM_ represent the refractive indices of the pSi monolayer and the polyNIPAM spheres in combination with surrounding medium, *L* the thicknesses of the respective layers, and *λ* the wavelength of the incident light. The terms *ρ*_a_, *ρ*_b_, and *ρ*_c_ describe the refractive index contrast between the different layers (Equation 5):

(5) $$\begin{array}{l} \rho_{a}=\left(n_{\mathrm{sol}}-n_{\mathrm{polyNIPAM}}\right)/\left(n_{\mathrm{sol}}+n_{\mathrm{polyNIPAM}}\right)\\ \rho_{b}=\left(n_{\mathrm{polyNIPAM}}-n_{\mathrm{pSi}}\right)/\left(n_{\mathrm{polyNIPAM}}+n_{\mathrm{pSi}}\right)\\ \rho_{c}=\left(n_{\mathrm{pSi}}-n_{\mathrm{Si}}\right)/\left(n_{\mathrm{pSi}}+n_{\mathrm{Si}}\right) \end{array}$$

where *n*_sol_, *n*_polyNIPAM_, *n*_pSi_, and *n*_Si_ are the refractive indices of the surrounding medium, the polyNIPAM layer, the porous silicon film, and silicon, respectively. However, the reflectance spectrum of our hybrid structures was similar in appearance to the reflectance spectrum of our reference sample, the pSi monolayer. Indeed, we observed a single peak in the FFT spectrum for our hybrid structure which corresponds to layer 2 (pSi film). This result is in accordance with studies on the deposition of lipid vesicles onto pSi layers monitored by RIFTS [24,25]. Presumably, the low refractive index of layer 1, composed of polyNIPAM spheres and surrounding solution, is responsible for the absence of the other two peaks in the FFT spectrum. In this context, it is important to note that the non-close packed arrangement of the polyNIPAM spheres leads to an effective refractive index of the top layer, which is composed of the refractive index of the polyNIPAM spheres and the surrounding medium. As the polyNIPAM spheres change their size and their refractive index upon swelling at the same time, the effective refractive index of this layer is rather complex. The deposition of a close packed monolayer of polyNIPAM spheres would reduce the complexity of this layer. In addition, the refractive index contrast between the pSi layer and the close packed polyNIPAM sphere layer would be smaller, leading to a more pronounced decrease in the FFT amplitude in comparison to pSi films decorated with a non-close packed layer of polyNIPAM spheres. However, our envisioned optical sensor shall utilize two different optical transduction methods, namely diffraction of light originating from the deposited non-close packed array of hydrogel microspheres and interference patterns resulting from light reflection at the interfaces of the porous silicon film. To obtain sufficient light diffraction from the hydrogel sphere monolayers, a non-close packed arrangement should be favorable.

**Figure 3:**
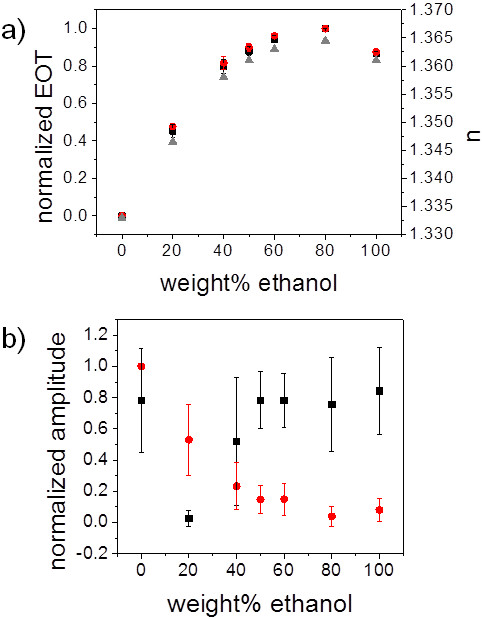
**Optical response of pSi monolayers with and without attached polyNIPAM microspheres to introduction of different ethanol/water mixtures. (a)** EOT changes of a pSi monolayer (red circles) and a pSi film covered with polyNIPAM microspheres (black squares). Refractive indices of ethanol/water mixtures for comparison (gray triangles). **(b)** Influence of polyNIPAM microspheres on the FFT amplitude of bare pSi films (red circles) and pSi layers covered with polyNIPAM microgel (black squares) which have been immersed in different solutions.

In Figure 3a, the EOT of a pSi monolayer decorated with polyNIPAM microspheres (black squares) and a bare pSi film (red circles) as a function of the weight% ethanol in the immersion medium are compared. The observed changes in the EOT demonstrate the infiltration of the solution into the porous layer and correspond to the refractive index changes in the ethanol/water mixtures. The refractive indices of the ethanol/water mixtures have been determined with an Abbé refractometer and are displayed as gray triangles in Figure 3a. However, the polyNIPAM microspheres on top of the pSi layer did not have an influence on the EOT of the porous film - as expected (black squares). In contrast, the amplitude of the FFT peaks changed differently for the two investigated structures (Figure 3b). Here, the amplitude of the FFT peak for a bare pSi monolayer depended solely on the refractive index of the immersion medium which dictates the refractive index contrast at the pSi surface. If polyNIPAM microspheres were bound to the pSi surface, the amplitude of the FFT peak reacted differently to immersion of the structure in alcohol/water mixtures with varying ethanol content. A distinct minimum in the amplitude of the FFT peak was observed in ethanol/water mixtures at 20 wt% ethanol content. This value coincides with published values for the collapse of polyNIPAM spheres in ethanol/water mixtures determined by DLS [23]. Hence, the decrease in the FFT amplitude could be explained by a decrease in the refractive index contrast at the pSi/polyNIPAM interface, which is based on the different refractive indices of the swollen (RI ~ 1.33) and collapsed polyNIPAM spheres (RI ~ 1.40) [26].

**Figure 4:**
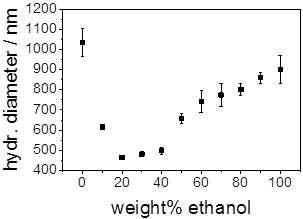
Hydrodynamic diameter of polyNIPAM microspheres in solution as function of ethanol content in alcohol/water mixtures determined by DLS.

**Figure 5:**
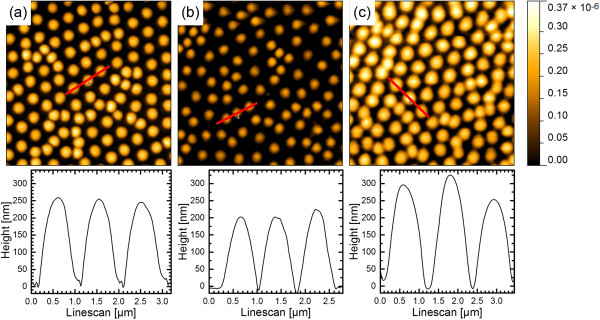
**AFM images of polyNIPAM microspheres attached to a pSi film in different surrounding media. (a)** In water, **(b)** in a mixture of ethanol and water containing 20 wt% ethanol, and **(c)** in ethanol. The bottom row shows the corresponding cross sections taken at the indicated red lines. AFM images size 10 × 10 μm.

Therefore, it stands to reason that the abrupt decrease in the FFT amplitude was caused by the deswelling of the polyNIPAM spheres attached to the pSi layer. To support this hypothesis, the diameter of the polyNIPAM microspheres in differently composed ethanol/water mixtures was determined using DLS (Figure 4). The polyNIPAM microspheres in solution showed the same trend for the deswelling in ethanol/water mixtures as the polyNIPAM microspheres which were deposited on the pSi layer. In both cases, the polyNIPAM microspheres collapsed to their minimum size at 20 wt% of ethanol. However, the reswelling of the polyNIPAM microspheres occurred considerably ‘slower’ in solution than for the surface-bound polyNIPAM microspheres if the ethanol content was further increased. This discrepancy could be related to the comparison of spherical polyNIPAM microgels in solution with polyNIPAM microspheres attached to a surface. In the latter case, the polyNIPAM has a hemispherical shape [27], and consequently, its density should differ from the dispersed hydrogel spheres. Thus, the swelling behavior of surface-bound polyNIPAM microspheres upon immersion in different media was studied using AFM (Figure 5). The AFM images show that the attached polyNIPAM microspheres were smaller than the same polyNIPAM microspheres in solution, in accordance to earlier studies [27]. In addition, the surface-bound polyNIPAM mcirospheres seemed to have almost the same size in pure ethanol and pure water in contrast to the DLS results. This observation was supported by extracting their heights from the AFM images which are summarized in Table 1. Hence, the AFM results suggest that the changes in the FFT amplitude of the pSi monolayer covered with a polyNIPAM microsphere array are indeed correlated to the shrinking and swelling of the hydrogel.

**Table 1:** Height of polyNIPAM microspheres bound to a pSi surface in different ethanol/water mixtures (determined by AFM)

**Ethanol/water mixtures, wt%/wt%**	**Height of adsorbed polyNIPAM microspheres in nm**
0:100	254 ± 83
20:80	196 ± 5
60:40	224 ± 24
100:0	292 ± 48

## Conclusions

To summarize, changes in the reflectance spectra of pSi monolayers, covered with a non-close packed array of polyNIPAM microspheres, upon immersion in different media were compared to the optical properties of untreated pSi films at the same conditions. The presence of the stimuli-responsive polyNIPAM microspheres led to distinct differences in the amount of reflected light from the pSi monolayer. By monitoring changes in the intensity of the reflected light, the swelling and shrinking of the polyNIPAM microspheres were successfully detected. As expected, the effective optical thickness of pSi monolayers and polyNIPAM covered pSi films reacted similarly upon immersion of the samples in ethanol/water mixtures. Future work will explore the detection of different biomolecules at the same time using the optical response of both the pSi film and the polyNIPAM microspheres.

## Competing interests

The authors declare that they have no competing interests.

## Authors’ contributions

MW determined the height of the polyNIPAM microspheres attached to the pSi surface using atomic force microscopy and in addition performed all DLS measurements. RFBV carried out all other experimental work including pSi etching, deposition of polyNIPAM spheres on pSi, collection of reflectance spectra, and SEM characterization. VA studied the reflectance spectra and provided value input for a better understanding of the optical data. CP conceived and designed the experiments and wrote the final version of the paper. All authors read and approved the final manuscript.

## Supplementary Material

Additional file 1: Figure S1 SEM images of porous silicon films decorated with polyNIPAM spheres. Click here for file
