# Supplementary material for: Optical characterization of porous silicon monolayers decorated with hydrogel microspheres
Source: Nanoscale Res Lett. 2014 Aug 22;9(1):425. doi: 10.1186/1556-276X-9-425 (PMC4151277; doi:10.1186/1556-276X-9-425)
Supplement: Additional file 1: Figure S1 — SEM images of porous silicon films decorated with polyNIPAM spheres. [file 1556-276X-9-425-S1.pdf]

ADDITIONAL INFORMATION FOR “Optical Characterization of porous Silicon Monolayers decorated with Hydrogel Microspheres”

R. F. Balderas-Valadez[1], M. Weiler[2,3], V. Agarwal[1] and C. Pacholski[2]

[1] CIICAp, UAEM, Av., Universidad 1001 Col. Chamilpa, Cuernavaca, 62210, Morelos, Mexico

[2] Max Planck Institute for Intelligent Systems, Department of New Materials and Biosystems, Heisenbergstr. 3, 70569 Stuttgart, Germany

[3] University of Heidelberg, Department of Biophysical Chemistry, Im Neuenheimer Feld 253, 69120 Heidelberg, Germany

[*] corresponding author: Pacholski@is.mpg.de

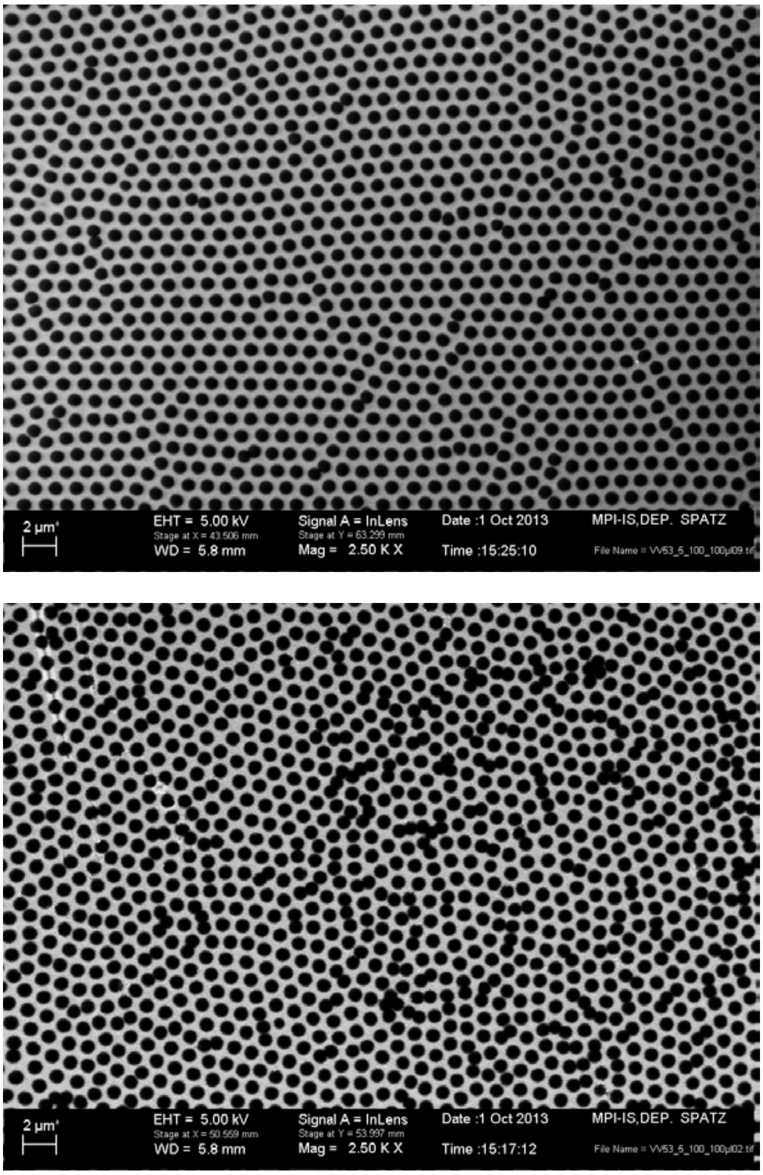

Figure S1: SEM images of porous silicon films decorated with polyNIPAM spheres.
